# Clinical utility of chitotriosidase enzyme activity in nephropathic cystinosis

**DOI:** 10.1186/s13023-014-0155-z

**Published:** 2014-11-19

**Authors:** Mohamed A Elmonem, Samuel H Makar, Lambertus van den Heuvel, Hanan Abdelaziz, Safaa M Abdelrahman, Xavier Bossuyt, Mirian C Janssen, Elisabeth AM Cornelissen, Dirk J Lefeber, Leo AB Joosten, Marwa M Nabhan, Fanny O Arcolino, Fayza A Hassan, Héloïse P Gaide Chevronnay, Neveen A Soliman, Elena Levtchenko

**Affiliations:** Department of Clinical and Chemical Pathology, Inherited Metabolic Disorder Laboratory (IMDL), Cairo University, Cairo, Egypt; Department of Pediatrics, Center of Pediatric Nephrology and Transplantation (CPNT), Cairo University, Cairo, Egypt; EGORD, Egyptian group of orphan renal diseases, Cairo, Egypt; Department of Pediatric Nephrology & Growth and Regeneration, University Hospital Leuven, Catholic University of Leuven, Leuven, Belgium; Department of Pediatric Nephrology, Radboud University Medical Center, Nijmegen, The Netherlands; University Hospitals Leuven & Department of Microbiology and Immunology, Laboratory Medicine, Catholic University of Leuven, Leuven, Belgium; Department of Internal Medicine, Radboud University Medical Center, Nijmegen, The Netherlands; Department of Neurology, Laboratory for Genetic, Endocrine and Metabolic Diseases, Radboud University Medical Center, Nijmegen, The Netherlands; CELL Unit, Université Catholique de Louvain & de Duve Institute, Bruxelles, Belgium

**Keywords:** Lysosomal storage disorders, Nephropathic cystinosis, Cystine crystals, Macrophage activation, Chitotriosidase enzyme, Clinical screening, Cysteamine, Therapeutic monitoring

## Abstract

**Background:**

Nephropathic cystinosis is an inherited autosomal recessive lysosomal storage disorder characterized by the pathological accumulation and crystallization of cystine inside different cell types. WBC cystine determination forms the basis for the diagnosis and therapeutic monitoring with the cystine depleting drug (cysteamine). The chitotriosidase enzyme is a human chitinase, produced by activated macrophages. Its elevation is documented in several lysosomal storage disorders. Although, about 6% of Caucasians have enzyme deficiency due to homozygosity of 24-bp duplication mutation in the chitotriosidase gene, it is currently established as a screening marker and therapeutic monitor for Gaucher’s disease.

**Methods:**

Plasma chitotriosidase activity was measured in 45 cystinotic patients, and compared with 87 healthy controls and 54 renal disease patients with different degrees of renal failure (CKD1-5). Chitotriosidase levels were also correlated with WBC cystine in 32 treated patients. Furthermore, we incubated control human macrophages *in-vitro* with different concentrations of cystine crystals and monitored the response of tumor necrosis factor-alpha (TNF-α) and chitotriosidase activity. We also compared plasma chitotriosidase activity in cystinotic knocked-out (n = 10) versus wild-type mice (n = 10).

**Results:**

Plasma chitotriosidase activity in cystinotic patients (0–3880, median 163 nmol/ml/h) was significantly elevated compared to healthy controls (0–90, median 18 nmol/ml/h) and to CKD patients (0–321, median 52 nmol/ml/h), P < 0.001 for both groups. Controls with decreased renal function had mild to moderate chitotriosidase elevations; however, their levels were significantly lower than in cystinotic patients with comparable degree of renal insufficiency. Chitotriosidase activity positively correlated with WBC cystine content for patients on cysteamine therapy (r = 0.8), P < 0.001. In culture, human control macrophages engulfed cystine crystals and released TNF-α into culture supernatant in a crystal concentration dependent manner. Chitotriosidase activity was also significantly increased in macrophage supernatant and cell-lysate. Furthermore, chitotriosidase activity was significantly higher in cystinotic knocked-out than in the wild-type mice, P = 0.003.

**Conclusions:**

This study indicates that cystine crystals are potent activators of human macrophages and that chitotriosidase activity is a useful marker for this activation and a promising clinical biomarker and therapeutic monitor for nephropathic cystinosis.

## Background

Nephropathic cystinosis (NC) is the prototype disorder for lysosomal transporter protein defects. *CTNS* gene mutations cause deficiency of cystinosin, the protein that transports cystine out of the lysosomes, resulting in the accumulation and crystallization of cystine in virtually all body cells. NC patients are asymptomatic at birth and usually develop normally during the first six months of life, where after they start to present with renal Fanconi syndrome. If not treated, renal failure invariably develops within the first decade of life [1].

The aminothiol cysteamine is the only specific treatment known today. It depletes lysosomal cystine through the formation of cysteine and the mixed disulfide cysteamine-cysteine complex which exits the lysosome via an intact PQLC2-transporter [2]. Cysteamine slows down the deterioration of the kidney function and postpones or even prevents extra-renal damage [1].

Elevated white blood cell (WBC) cystine concentration is the diagnostic cornerstone, as the pathognomonic corneal cystine crystals can frequently be missed in children younger than 18 months and genetic testing usually takes a longer time [3]. WBC cystine assay was introduced by Oshima et al. who used [^14^C] cystine and E.coli cystine-binding protein [4]. Currently most laboratories switched to HPLC [5] or LC-MS/MS [6] methods to avoid working with radioactivity. While biochemical cystine determination can be well-standardized, the major source of imprecision is attributed to the WBC isolation and storage [7]. Furthermore, blood for cystine assay must be taken six hours after the last cysteamine dose which is not always clinically feasible. Finally, it is currently unknown whether WBC cystine levels adequately represent cystine accumulation in tissues.

Chitotriosidase was first associated with marked lysosomal storage in Gaucher’s disease patients [8]. Later its elevation was detected in many other lysosomal storage disorders (LSDs) [9] and also in multiple other diseases as β-thalassemia [10] and sarcoidosis [11].

Due to the absence of its natural substrate (chitin) in mammals and its secretion mainly by activated macrophages, chitotriosidase was rapidly linked to innate immunity as a defense mechanism against chitin coated pathogens [12]. However, its elevation in response to storage and chronic inflammatory disorders clarified its other nature as a marker of macrophage activation.

Despite the fact that about 6% of Caucasians have complete enzyme deficiency due to homozygosity to the 24-bp duplication mutation at exon 10 of the chitotriosidase gene *(CHIT1)* [13], plasma chitotriosidase activity has been established as a screening marker and therapeutic monitor for enzyme replacement therapy in Gaucher’s disease [14]. Further studies reported the validity of the clinical use of chitotriosidase activity in the screening of other lysosomal disorders as Niemann-Pick disease and Gangliosidosis M1 [15] and for therapeutic monitoring of male Fabry patients [16]. Recently, chitotriosidase elevation was documented in a case-report of nephropathic cystinosis [17], and it was suggested that it could be of value in monitoring cysteamine therapy.

In the current study, we aimed to evaluate the potential of plasma chitotriosidase activity for the clinical screening and therapeutic monitoring of NC patients. As a proof of principle we tested our hypothesis on the cellular level in control human macrophages and in the plasma of the murine cystinotic model.

## Methods

### Patients

Forty five NC patients were recruited over the period: November 2010- April 2013. Twenty three Egyptian patients (10 m-16y, median 3.8y) were recruited at the Center of Pediatric Nephrology and Transplantation (CPNT), Cairo University Children’s Hospital, Egypt and 22 European patients (2-49y, median 26.5y) were recruited at Pediatric Nephrology Clinics, University Hospital Leuven, K.U. Leuven, Belgium and Pediatric Nephrology and Internal Medicine departments, Radboud University Medical Center, Nijmegen, the Netherlands.

Nine Egyptian patients were newly-diagnosed (10-28 m), while the rest of Egyptian and all European patients were already on cysteamine therapy. Chitotriosidase activities in cystinotic patients were compared to 54 healthy pediatric controls (1-16y, median 4.8y), 33 healthy adult controls (22-48y, median 36y), 24 renal pediatric controls: 12 with renal Fanconi syndrome after excluding NC (2-5y, median 3y) with chronic kidney disease (CKD 1-4) and 12 with end stage renal disease (ESRD, CKD 5) due to a variety of other causes (2-12y, median 6y), 15 patients with diabetic nephropathy (CKD 1–4, 26-46y, median 32y) and 15 patients with ESRD (CKD 5, 19-58y, median 41y). The current study was approved by the Institutional Research and Ethical Committee and written informed consents were obtained from healthy controls and patients or legal guardians in case of minors.

### *Ctns* knocked-out mice

Blood was collected from the retro-orbital plexus of ten C57BL/6 C*tns(−/−)* mice (12-15 m, median 13 m) and ten wild-type mice (12-15 m, median 12 m) with heparinized capillaries. Blood was centrifuged 20 min at 5000 rpm and plasma was kept at −80°C till assayed.

### L-cystine crystal preparation

120 mg of L-cystine (Fluca) was dissolved in 200 ml of distilled water through boiling while stirring. Solution was left to stand overnight. After centrifugation, supernatant was transferred to another container as fully saturated cystine solution, while the crystallized deposit was kept in 5 ml of saturated solution. After vigorous mixing 0.5 ml was transferred and dried in a vacuum centrifuge. According to the pellet’s weight the crystal concentration was adjusted to 10 mg/ml. Crystal size was mostly below 10 μm. Cystine saturated and crystal stock solutions were kept at 4°C till used.

### Monocyte maturation

Heparinized blood was collected from healthy donors. Monocytes were separated with CD14 magnetic micro-beads according to the supplier’s protocol (MACS, Miltenyi Biotec) and incubated in RPMI-1640 (Lonza) supplemented with 10% FCS, 2 mM L-glutamine and 50 μg/ml gentamycin. Cells were seeded at a concentration of 1-2million/ml, while adding 0.1 μg/ml GM-CSF (Gentaur) and 50 nmol/ml β-mercaptoethanol. After 3 days half amount of the culture medium was replaced with fresh medium containing GM-CSF (0.2 μg/ml) and β-mercaptoethanol (50 nmol/ml). After another four days differentiated macrophages were harvested with trypsin (170 U/ml) + EDTA (200 mg/L) (Lonza) and immediately used.

### Macrophage exposure to cystine crystals

Mature macrophages were seeded in 25 cm^2^ flasks at a density of 100,000 cells/ml, left to stand for 2 hours, then washed with PBS to eliminate non-adherent cells. Cystine crystals were washed three times in cystine saturated solution. Supernatant was discarded and cystine saturated culture medium was added (Culture stock solution with crystal concentration 10 mg/ml), then two experiments were performed:

1. There was no previous data available for the suitable amount of cystine crystals to stimulate macrophages, therefore we adapted a wide concentration range (0–200 ug/cm^2^) guided by similar experiments with other crystals as calcium oxalate [18]. Cystine crystal stock solution was mixed and transferred to fresh medium in concentrations ranging from 0 to 200 μg/cm^2^ (0, 1, 5, 25, 100 and 200 μg/cm^2^), a triplicate of each concentration was performed in two separate experiments. Adherent macrophages were incubated with cystine crystals at 37°C, 5% CO2 for three hours, and then washed with PBS to eliminate excess crystals and fresh medium was added. At determined intervals from the crystal elimination (0, 4, 8, 24 and 48 hours) 300 μl of culture fluid was sampled, centrifuged and supernatant kept at −80°C for TNF-α and chitotriosidase measurements. Resulting values were corrected for medium volume changes.
2. Adherent macrophages were incubated with crystal solution at a fixed concentration of 100 μg/cm^2^, and then washed as previously described. At determined intervals: 0, 8, 24, 48 and 72 hours cells were harvested in duplicate. In the same experiment control macrophages were harvested at the same intervals. All pellets were kept immediately at −80°C till assaying chitotriosidase activity.

### Microscopy

Macrophages were visualized directly and after 48 hours by inverted phase contrast microscopy within culture flasks after incubation and washing excess crystals (DM-IL-LED, Leica microsystems).

### TNF-α immunoreactivity

TNF-α was measured in culture supernatant by a sandwich ELISA technique according to the manufacturer’s protocol (BD Pharmingen).

### Chitotriosidase enzyme activity

Either plasma, culture supernatant or macrophage cell-lysate were used as samples. Macrophage lysis buffer included 1% IGEPAL CA-630, 1% SDS, 12 mM Na deoxycholate, 0.6 mM PMSF, 1 μg/ml aprotinin and 1 mM Na orthovanadate (Sigma). Activity was measured as previously described [8]. Results were expressed as nmol/ml/h for plasma and nmol/mg pellet protein/h for supernatant and cell-lysate.

### Chitotriosidase genotype

Being a common mutation in many ethnicities and the main cause of chitotriosidase deficiency worldwide [13], DNA analysis for detecting the 24-bp duplication mutation within exon ten of *CHIT1* gene was performed for cystinotic patients. PCR was performed as previously described [13] resulting in the formation of a 75-bp band in the wild-type, a 99-bp band in the homozygously mutated and both in the heterozygous. Healthy controls for macrophage experiments were confirmed as being wild-type.

### WBC cystine assay

Ten ml of blood was collected from NC patients. WBC were separated using dextran 3%, and pellets were kept at −80°C with 150 μl of N-ethylmaleimide and 50 μl of 12% sulfosalicylic acid till assayed. Cystine was quantified by LC-MS/MS (Micromass, Waters) as previously described [6].

### Statistical analysis

Unless otherwise stated all results were expressed as range and median. Differences were analyzed using a two-tailed Mann–Whitney U test with the Finner adjustment for multiple testing [WinPepi statistical software package]. Pearson correlation coefficient (r) was used for testing correlations and P < 0.05 was set as the level of significance.

## Results

### Plasma chitotriosidase activity

Chitotriosidase levels in 45 cystinotic patients ranged from 0 to 3880 (median 163 nmol/ml/h), which were significantly different from 87 healthy controls ranging from 0 to 90 (median 18 nmol/ml/h), P < 0.001, including 54 healthy pediatric controls ranging from 0 to 72 (median 14 nmol/ml/h) and 33 healthy adult controls ranging from 0 to 90 (median 31 nmol/ml/h). Levels in the cystinotic patients were also significantly different from the 54 CKD controls ranging from 0 to 321 (median 52 nmol/ml/h), P < 0.001. Renal controls consisted of 24 pediatric patients ranging from 2 to 144 (median 39 nmol/ml/h) and 30 ranging from 0 to 321 (median 73 nmol/ml/h). Table 1 summarizes chit otriosidase values for different subgroups of patients and controls. Poorly controlled cystinotic patients with ESRD demonstrated very high chitotriosidase values (899, 962, 1346, 1487 and 3880 nmol/ml/h), P = 0.002 compared to the ESRD control group.

**Table 1:** **Chitotriosidase activity in control groups and cystinotic patients**

**Group**	**N**	**Age in years range (median)**	**Chitotriosidase activity nmol/ml plasma/h range (median)**	**P values**
All normal controls	87	1-48 (12)	0-90 (18)	
Pediatric normal controls	54	1-16 (4.8)	0-72 (14)	
Adult normal controls	33	22-48 (36)	0-90 (31)	<0.001 with pediatric normal controls
All renal controls	54	2-58 (22)	0-321 (52)	<0.001 with all normal controls
Pediatric renal controls	24	2-12 (4.6)	2-144 (39)	<0.001 with pediatric normal controls
Fanconi syndrome	12	2-5 (3)	5-44 (19)	0.60 with pediatric normal controls
ESRD	12	2-12 (6)	2-144 (67)	<0.001 with pediatric normal controls
				<0.001 with pediatric Fanconi syndrome
Adult renal controls	30	19-58 (38)	0-321 (73)	<0.001 with adult normal controls
Microalbuminuria	15	26-46 (32)	8-143 (60)	0.008 with adult normal controls
ESRD	15	19-58 (41)	0-321 (87)	<0.001 with adult normal controls
				0.40 with diabetic nephropathy
All controls with ESRD	27	2-58 (22)	0-321 (75)	
All controls below 5 years	39	0.5- 4.5 (2.2)	0-49 (15)	
Cystinosis patients	45	0.8-49 (13)	0-3880 (163)	<0.001 with all normal controls
				<0.001 with all renal controls
All pediatric patients	25	0.8-16 (3.8)	0-3880 (268)	<0.001 with pediatric normal controls
				<0.001 with pediatric renal controls
All adult patients	20	18-49 (28)	0-1487 (78)	<0.001 with adult normal controls
				0.44 with adult renal controls
Cysteamine treated patients				
Cystine* <1	11	17-49 (34)	18-342 (52)	0.71 with all renal controls
Cystine* >1 and <4	13	2-38 (24)	0-843 (245)	0.006 with all renal controls
Cystine* >4	12	3.5-36 (10.5)	84-1487 (788)	<0.001 with all renal controls
Cystinosis patients with ESRD	5	8-24 (12)	899-3880 (1346)	0.002 with all controls with ESRD
Newly diagnosed patients	9	0.8-2.7 (1.6)	0-288 (122)	<0.001 with all controls <5 years

*WBC cystine in nmol ½ cystine/mg protein; ESRD, end stage renal disease.

**Figure 1:**
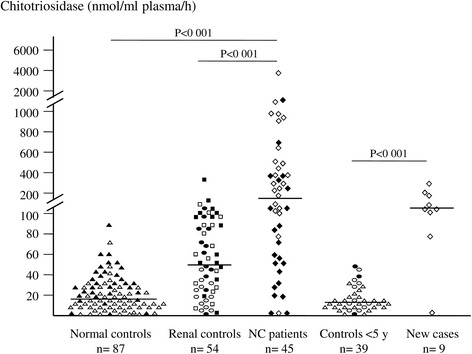
**Plasma Chitotriosidase activities in different test groups. Normal pediatric controls (∆), normal adult controls (▲), pediatric controls with renal Fanconi syndrome (○), pediatric controls with ESRD (●), adult renal controls with microalbuminuria (□), adult renal controls with ESRD (∎** **) and Cystinotic patients; European (♦) and Egyptian (◊).** Forty five cystinotic patients were compared to 54 healthy pediatric controls, 33 healthy adult controls, 24 renal disease pediatric patients (12 with Fanconi syndrome and 12 with ESRD) and 30 renal disease adult patients (15 with albuminuria and 15 with ESRD). Nine newly diagnosed Egyptian patients were compared to all controls below 5 years of age (26 normal and 13 renal). Averages of duplicates were used. Horizontal bars represent the median value for each group.

Among our series, nine cystinotic patients were newly-diagnosed. Their chitotriosidase activity ranged from 0 to 288 (median 122 nmol/ml/h), while their age matching healthy and CKD controls ranged from 0 to 49 (median 15 nmol/ml/h), P < 0.001 (Figure 1).

**Figure 2:**
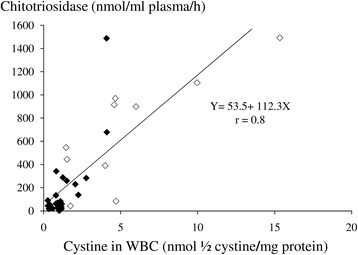
**Correlation of plasma chitotriosidase activity with WBC cystine assay in 32 cystinotic patients, 22 Europeans (◊) and 10 Egyptians (◊), all on different doses of cysteamine therapy.** Four treated Egyptian patients were not sampled for WBC cystine at the same time of the chitotriosidase sampling, so they were not included in the analysis. Pearson correlation coefficient was used.

Chitotriosidase levels were generally higher in the 23 Egyptian patients ranging from 0 to 3880 (median 288 nmol/ml/h) compared to 22 European patients, in whom they ranged from 0 to 1487 (median 70 nmol/ml/h). On the other hand, WBC cystine levels in the ten measured Egyptian patients on treatment ranged from 1.5 to 15.3 (median 4.6 nmol ½ cystine/mg protein), while in the 22 European patients it ranged from 0.27 to 4.1 (median 1.1 nmol ½ cystine/mg protein), r = 0.8 and 0.78 with corresponding chitotriosidase values, respectively. Combined Pearson correlation coefficient was r = 0.8, P < 0.001 (Figure 2).

Over the last year we further evaluated a naïve set of suspected patients (n = 13) who were referred to our laboratory for WBC cystine assay. WBC cystine levels for nine negative cases ranged from 0.06 to 0.36 nmol ½ cystine/mg protein, while their chitotriosidase activities ranged from 7 to 43 nmol/ml/h. As for the four positive cases their cystine levels ranged from 3.9 to 12.3 nmol ½ cystine/mg protein, and their chitotriosidase activities ranged from 76 to 373 nmol/ml/h.

### Chitotriosidase genotype

Thirty six patients were analyzed for the presence of the 24-bp duplication. We found 21 wild-type, 13 heterozygous and two homozygous for the mutation. Three patients were below detection limit for chitotriosidase in our study, two of them were homozygous for the mutation (one Egyptian and one European) and the third (a 2 year’s old treated European child) was heterozygous (Figure 1).

### Macrophage activation by cystine crystals

**Figure 3:**
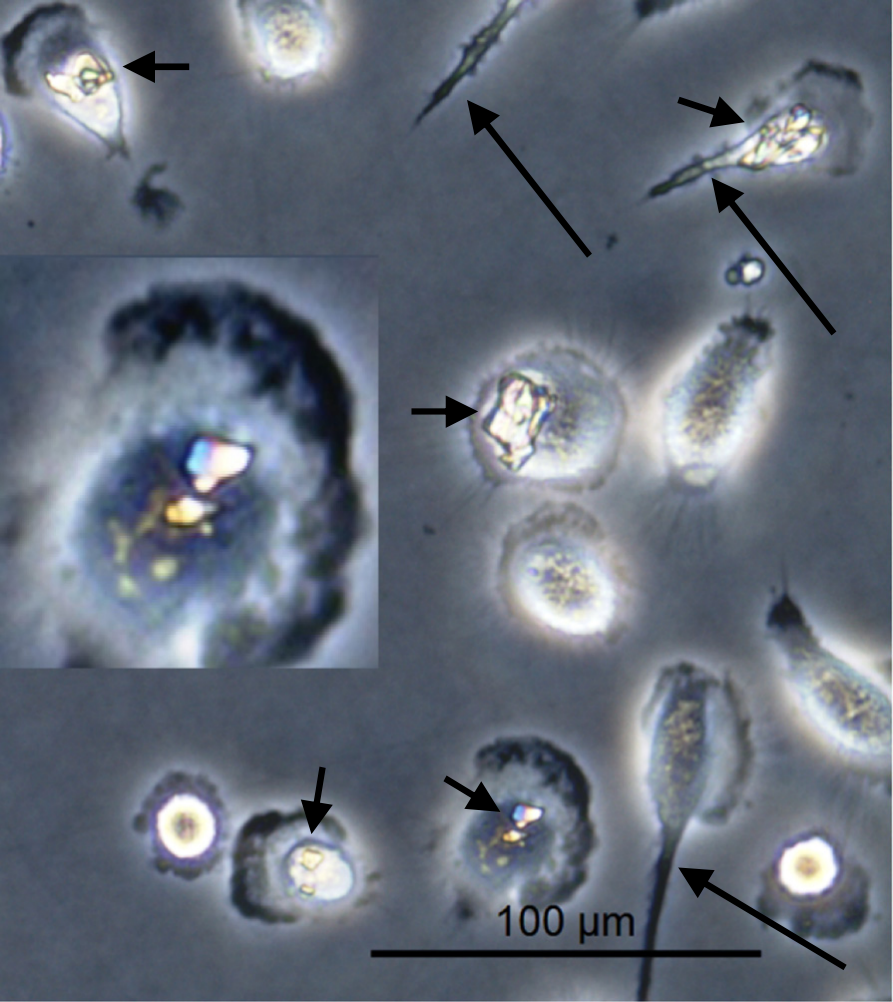
**A phase contrast micrograph of macrophages in culture after exposure to cystine crystals.** Many macrophages show intracellular crystals (short arrows) and some show pseudopodia (long arrows) to help in movement and engulfment of the crystals.

**Figure 4:**
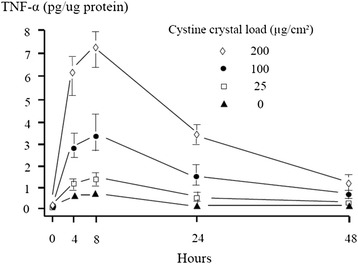
**TNF-α concentrations in the supernatant of macrophage cell culture after incubation with different concentrations of cystine crystals (experiment 1).** A triplicate of each concentration was performed in two separate experiments. Concentrations were expressed as pg/μg protein of macrophage pellets. Horizontal bars represent the range of TNF-α values for each crystal concentration.

Uptake of cystine crystals by mature macrophages was demonstrated by phase contrast microscopy (Figure 3). Over 95% of macrophages were still strongly attached to the culture flask surface after 48 hours of crystal elimination denoting cell viability. Looking for direct evidence of macrophage activation after crystal uptake, TNF-α was measured at predetermined intervals. The detected peak level for TNF-α was eight hours after crystal elimination, and the maximum response was obtained with the highest crystal concentration used (200 μg/cm^2^), which was about ten folds the zero level response (Figure 4).

### Chitotriosidase activity in macrophage cell culture

**Figure 5:**
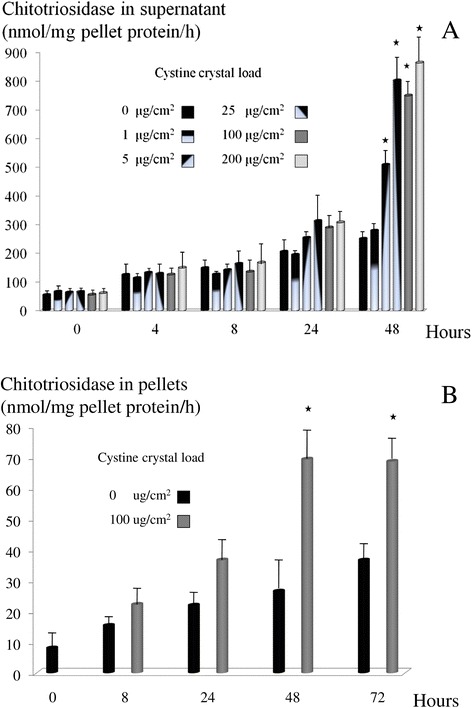
**Chitotriosidase activity in the supernatant and cell-lysate of macrophages after incubation with cystine crystals. (A)** Chitotriosidase activity in the supernatant of macrophages incubated with different concentrations of cystine crystals (experiment 1). **(B)** Chitotriosidase activity in the cell-lysate of macrophages incubated with 100 ug/cm^2^ cystine crystals (experiment 2). Results were represented as averages and standard deviations of 2 independent experiments in triplicate for experiment 1 and in duplicate for experiment 2. Enzyme activities for both experiments were expressed as nmol enzyme activity/mg pellet protein/h. *represents P < 0.05 with the zero level crystal concentration.

Chitotriosidase activity was increased in the supernatant and cell-lysate of macrophages incubated with cystine crystals (Figure 5A and B). Activity in the supernatant was higher than in cell-lysate when referred to pellet’s protein content indicating the enzyme rapid extracellular release. Chitotriosidase activities were significantly different from zero level crystal concentration at 48 hours for most concentrations tested, however a trend was observed already after 24 hours.

### Chitotriosidase activity in mice

In an attempt to consolidate our hypothesis in a different species, we found a significant difference between plasma chitotriosidase levels in C*tns(−/−)* mice (100–198, median 137 nmol/ml/h, n = 10) and C*tns(+/+)* mice (61–82, median 73 nmol/ml/h, n = 10), P = 0.003.

## Discussion

Macrophage activation has been previously reported in many LSDs. Although exact mechanisms are largely unknown, this might reflect the altering signaling pathways in response to storage materials [19]. Furthermore, different types of crystals such as calcium oxalate [18] and monosodium urate [20] were shown to stimulate human macrophages. Tissue macrophages harboring cystine crystals have been demonstrated in cystinotic patients long ago [21,22]. Several immune enhancing molecules such as interferon-gamma, TNF-α and lipopolysaccharide have all been shown to stimulate chitotriosidase expression in human macrophages [23]. We hypothesized that in analogy to other crystal types cystine crystals are not inert structures; they tend to activate macrophages and as a consequence of the elicited activation, chitotriosidase release can be detected clinically and confirmed experimentally.

In our study, apart from a single newly-diagnosed cystinotic patient homozygous for the chitotriosidase deficiency mutation, there was no overlap in chitotriosidase values between the other twelve newly-diagnosed (8 in the study group and 4 naïve patients) and age-matched controls (39 in the study group and 9 naïve patients). This indicates that chitotriosidase can be a helpful guide in suspected individuals when WBC cystine measurement is not immediately available, at least to prioritize patients as high or low risk till properly diagnosed.

Many poorly controlled cystinotic patients achieved very high chitotriosidase activities (Figure 1); the highest value (3880 nmol/ml/h, 216 folds the median value of normal controls) was observed in an eight years old Egyptian boy with ESRD who died shortly after this measurement. The median chitotriosidase value in the 45 cystinotic patients was about 10 folds the median of normal controls. Apart from Gaucher’s disease which has extremely high chitotriosidase values few other LSDs achieve moderately high values comparable to cystinosis, especially sphingolipidoses as Niemann-Pick, GM1 and Krabbe [15,24].

European patients were generally much better therapeutically controlled than Egyptian patients. There was no major difference in the severity of responsible mutations as most mutations were truncating in both cohorts; however, this difference in therapeutic response might be due to the delayed diagnosis and suboptimal therapeutic doses of cysteamine in Egyptian patients given the financial and logistical constraints in providing this orphan drug in a developing country [25]. This was evident from WBC cystine levels in each group. As it could be expected, chitotriosidase activities were much lower in European patients in accordance with the proposed role of chitotriosidase as an alternative therapeutic monitor.

When compared to WBC cystine, plasma chitotriosidase is much easier, faster, more economic, stable and needs a much smaller sample (1 ml or less) which is more convenient in young children. Furthermore, WBC cystine is not the perfect measure of therapeutic response, as polymorphonuclear leucocytes harboring cystine are short living cells (≈12 hours); therefore, WBC cystine represents relatively a short time of therapeutic control. Hypothetically, if a patient complies strictly with cysteamine treatment a few days before the assay, he may appear properly controlled regardless of previous compliance status. Chitotriosidase, on the other hand, is produced by macrophages which are longer living cells (months to years), and therefore should provide a better notion about therapeutic response over a longer time period. For confirming this statement a more extended longitudinal study is currently underway to better correlate clinical course under cysteamine therapy with chitotriosidase levels.

In newly-diagnosed patients, although chitotriosidase levels were significantly higher than age-matched controls; there was a poor correlation with WBC cystine in cases diagnosed below two years of age. This could be explained by the relative immaturity of human macrophages in infants and young children [26] and better preservation of kidney function, therefore necessitating cautious interpretation of chitotriosidase levels in this age group.

Two of our patients (2/45 or 4.4%) were homozygous for the 24-bp duplication mutation in accordance with the previously reported prevalence [13]. The third patient having values below the detection limit (A properly controlled 2 years old European child) was heterozygous. The second sample taken few months later showed higher level of chitotriosidase (within the enzyme detectable range) that also corresponded with a higher WBC cystine value.

We have to stress here that chitotriosidase activity will not replace the WBC cystine assay in NC diagnosis; however, we believe that it is a useful clinical screening test and a promising therapeutic monitor. Concerns about false negative screening when the *CHIT1* gene is homozygously mutated can be met with setting a lower cut-off limit for the assay, below which the chitotriosidase result is considered of no value and the suspected patient should proceed anyway to WBC cystine assay or to molecular diagnosis.

Although three of our newly diagnosed patients were heterozygous for the *CHIT1* mutation, their plasma chitotriosidase activities (78, 122 and 138 nmol/ml/h) were clearly above their age-matched controls (0–49 nmol/ml/h, n = 39). Furthermore, recent evidence in Gaucher’s disease implies that chitotriosidase activities in the heterozygous patients, although significantly decreased compared to the wild-type, respond to treatment in a very similar way. Chitotriosidase activities in the wild type Gaucher’s patients after one year of starting enzyme replacement therapy decreased by 79 ± 13% of base line values, while in a similar cohort of heterozygous patients the percent of decrease was also similar 77 ± 16% [27]. Likewise, Gaucher patients with the common *CHIT1* polymorphism p.G102S were reported to have a similar decline in chitotriosidase activity in response to optimal enzyme replacement therapy (72 ± 18% in the heterozygous and 76 ± 20% in the homozygous) [27].

We noticed significant elevations in chitotriosidase activity in control subjects with ESRD compared to normal pediatric and adult controls (P < 0.001). This could be explained by the fact that macrophage infiltration of renal tissue is a common feature of most human chronic kidney disease and that pathogenic M1 subpopulations are implicated in the pathology of renal fibrosis [28]. However, chitotriosidase levels in ESRD controls were still much lower compared to NC patients with the same degree of renal disease because macrophage activation in cystinosis is a global pathology and not just restricted to the kidney.

There are two possible mechanisms of macrophage activation by cystine in cystinotic patients. The first is the gradual accumulation of cystine inside macrophages up to crystallization because macrophages also have cystinosin defect and cannot get rid of their lysosomal cystine similar to other cell types. The second mechanism is the engulfment of dying and apoptotic cells in different tissues with ready formed cystine crystals inside or around these cells and because macrophages have the same pathology they cannot dissolve these phagocytized crystals.

We tried to address both mechanisms initially and used cystine dimethyl ester (CDME) as a rapid loading molecule of cystine into cultured macrophages; however, this molecule was toxic to macrophages and we couldn’t achieve our target with non-toxic levels. Using patients macrophages was not feasible because of the high amount of blood required for the experiments. Thus, we addressed the second mechanism through the direct incubation of control human macrophages with different concentrations of cystine crystals *in-vitro* and succeeded in demonstrating the phagocytosis of cystine crystals, the activation of macrophages only by the crystals and the release of chitotriosidase enzyme.

TNF-α, being an established marker of macrophage activation and a direct stimulant of chitotriosidase release [23], was measured sequentially in the supernatant of macrophages exposed to cystine crystals. TNF-α quantified response was correlated with the crystal concentration used (Figure 4), denoting the evident activation of macrophages upon exposure and engulfment of crystals. Macrophage activation was further confirmed through the elevation of chitotriosidase activity in both supernatant and cell-lysate. In fact, this was demonstrated in control macrophages that were capable of processing cystine, while *in-vivo* this stimulation might be even more pronounced in cystinotic macrophages.

Recently, Principe et al. attributed NC with the inflammasome system activation and argued that the inflammation caused by cystine crystals could be responsible for the rapid progression to ESRD in cystinosis unlike other types of hereditary Fanconi syndromes [29]. Similar observation was made by Knauf et al. who demonstrated the importance of the inflammasome mediator NALP3 in the renal injury and fatality of mice with oxalate nephropathy [30]. Okamura et al. further elucidated the role of cysteamine treatment in reducing macrophage accumulation in the renal interstitium of mice exposed to chronic renal injury and reducing the generation of reactive oxygen species in these macrophages [31]. Thus, the clinical assessment of macrophage activation in NC as an indicator of disease activity and/or therapeutic response seems to be a promising approach. Measuring chitotriosidase activity in that regard has many advantages compared to other circulating cytokines, especially that the enzyme is long-living and very stable, thus, less affected by acute infections or immunosuppressive drugs and it can be also measured in blood spots [32].

The significant elevation of plasma chitotriosidase in *Ctns(−/−)* mice indicates that its correlation with cystinosis is not species specific. Although the median value for the diseased was only double that of the wild-type, this might refer to the shorter duration of the pathological process and the relatively lower differential number of activated macrophages in mice [33].

The recent successful treatment of the murine cystinotic model by bone marrow cell transplantation [34] and hematopoietic stem cell gene therapy [35], if proven successful in human patients, will have a powerful impact on the disease therapeutic strategy. However, the problem on how to evaluate the patient’s response post-transplantation is not yet solved as estimating cystine accumulation in tissue biopsies is too invasive for routine follow-up and measuring cystine in the donor’s WBCs is useless. Chitotriosidase, on the other hand, is rapid and non-invasive. When compared to a base-line level it would represent the activity of the recipient’s tissue macrophages and would be a reasonable indicator of total body cystine load. Notably, chitotriosidase enzyme was reported to show significant declines in response to successful bone marrow transplantation in Gaucher’s disease [14] and in β-thalassemia major [36].

## Conclusions

In conclusion, plasma chitotriosidase activity was significantly elevated in cystinotic patients at diagnosis over age matched normal and renal controls and correlated with WBC cystine assay for treated patients above 2 years of age. Thus, chitotriosidase is a promising clinical biomarker and therapeutic monitor in nephropathic cystinosis patients.
